# Species associations and distributions of soil entomopathogenic fungi *Metarhizium* spp. in Japan

**DOI:** 10.1080/21501203.2017.1386244

**Published:** 2017-10-10

**Authors:** Oumi Nishi, Kazuhiro Iiyama, Chisa Yasunaga-Aoki, Susumu Shimizu

**Affiliations:** a Forest Entomology Division, Forestry and Forest Products Research Institute, Tsukuba City, Japan; b Research Fellowship for Young Scientists, Japan Society for the Promotion of Science, Chiyoda-ku, Tokyo, Japan; c Laboratory of Insect Pathology and Microbial Control, Institute of Biological Control, Kyushu University, Higashi-ku, Fukuoka City, Japan; d Nishi-Nippon Junior College, Chuo-ku, Fukuoka City, Japan

**Keywords:** Entomopathogenic fungi, *Metarhizium*, species associations, habitat preference, PCR-RFLP

## Abstract

*Metarhizium* Sorokīn (Hypocreales: Clavicipitaceae) is a genus of facultative parasites of insects found in soils from various environments and is used for pest management. Understanding the habitat selection of *Metarhizium* spp. is critical to improve the efficacy and persistence of these fungi as microbial insecticides. This study sought to determine the habitat preferences of *Metarhizium* spp. in Japan. We identified 302 isolates of *Metarhizium* spp. as eight species by a combination of PCR–RFLP and phylogenetic analysis of DNA sequences. *M. pingshaense* was the predominant species in Japan and was most frequently isolated from both forest and agricultural environments. On the other hand, *M. brunneum* and *M. pemphigi* were comparatively restricted to forest environments. A similar species association was detected in a small area that was intensively investigated, where 7 species including 14 genotypes were isolated from soil. The results of this study have revealed different habitat preferences among *Metarhizium* spp. in Japan.

## Introduction

*Metarhizium* Sorokīn is one of the most commercially important entomopathogenic fungi, and some isolates of this genus have been used as biological control in pest management against locusts, termites, spittlebugs, and white grubs (Zimmermann 1993). *M. anisopliae sensu lato* (s. l.), the most common group in this genus, has been detected in soils from various environments and has been isolated from over 200 species of insects (Zimmermann 1993).

*M. anisopliae* s. l. is known to be a complex of cryptic species which are now classified by a molecular phylogenetic approach and can be distinguished by DNA sequences (Bischoff et al. 2009). Bidochka et al. (2001) demonstrated that cryptic species of *M. anisopliae* s. l. were associated with specific habitat type preferences rather than host insects. They showed that the genotypic groups of *M. anisopliae* s. l. in eastern Canada were linked to habitat type rather than to insect hosts; isolates from open field habitats (OG1) belonged to a different genetic group compared with those from forest soil samples (OG2). The two groups also showed opposite physiological growth characteristics, which appeared to be an adaptation to the temperatures and UV radiations of their habitats. One isolates each from OG1 and OG2 were later identified as a different species, *M. robertsii* (*Mr*) and *M. brunneum* (*Mb*), respectively, by the recent taxonomy of *Metarhizium* (Bischoff et al. 2009). Furthermore, *Mr* and *Mb* in the same area were preferentially detected from the rhizospheres of wildflowers and trees, respectively (Wyrebek et al. 2011).

Characterizations of the habitat preferences of entomopathogenic fungal species appear to be important for selecting suitable strains for pest control. This is because, particular entomopathogenic fungal species will adapt to the abiotic conditions of their specific habitat so as to persist in that habitat, as seen in the case of *M. anisopliae* s. l. in eastern Canada (Bidochka et al. 2001). Fungal strains that exhibit good persistence in the introduced environments will have a higher probability of contact with a sufficient quantity of propagules to cause disease and will therefore be effective in pest management.

The habitat preferences of species or genotypic groups of *Metarhizium* spp. have been studied in some countries. Although *Mb* was restricted to forest areas in eastern Canada, in Denmark, *Mb* was predominant in an agricultural field (Steinwender et al. 2011: Wyrebek et al. 2011). The associations of genotypes and habitat types have been studied for *M. anisopliae* s. l. in Chile, Turkey, and western Canada; however, clear associations have not been observed (Velasquez et al. 2007; Inglis et al. 2008; Sevim et al. 2012). *Metarhizium* spp. in Asia have rarely been studied, especially in terms of habitat preferences.

In a previous study, soil isolates of *Metarhizium* spp. in Japan were identified as eight species (Nishi et al. 2011). These species were significantly associated with *in vitro* germination characteristics of conidia under high and low temperatures (Nishi et al. 2013). However, habitat preference of these isolates was not sufficiently investigated. In this study, we analysed the association of species with habitat types with additional isolates of *Metarhizium* spp. using a newly developed genotypic marker for fast genotypic grouping.

## Materials and methods

### Soil samples

A total of 197 soil samples were collected from various environments in Japan from April 2008 to October 2013 (Table 1). An additional 82 samples were collected in an area around Mt. Tachibana-yama (Fukuoka, Japan) for intensive investigation of *Metarhizium* in a small area (approximately 1.6 × 1.0 km) (Table 1). Soil samples (approximately 5–10 cm deep) were collected after removal of surface litter. All soil samples were placed in plastic bags and stored at 4°C before use.

**Table 1.** Number of soil samples and isolates of *Metarhizium* spp. from different locations.

Locations (Latitude N°)	Soil samples				Isolates
	Total	Forest or Wood	Cultural field	Others	
45–46	7	0	1	6	3
43–44	16	7	2	7	11
42–43	5	3	2	0	6
40–41	2	1	1	0	1
38–39	2	0	0	2	1
35–36	14	2	2	10	22
34–35	3	0	0	3	7
33–34	58	20	15	23	45
32–33	36	14	10	12	26
31–32	31	14	11	6	39
30–31	10	8	1	1	12
28–29	4	0	1	3	2
26–27	9	4	0	5	15
Total	197	73	46	78	190
(Detection rate (%))	(65.5)	(67.1)	(60.9)	(66.7)	
Mt. tachibana-yama area	82	38	9	35	112
(Detection rate (%))	(78.9)	(92.1)	(88.9)	(62.9)	

### Isolation of *Metarhizium* from soil

Each soil sample was suspended in a sterile aqueous solution of 0.05% Tween 80 and thoroughly shaken. Each sample was then serially diluted, and each dilution was then spread-plated (100 µl) onto a selective agar medium (60 g l^−1^ oatmeal flour, 12.5 g l^−1^ agar, 0.3 g l^−1^ chloramphenicol, 1.0 g l^−1^ cycloheximide), a simplified medium of Yaginuma and Takagi (1986). Cultures were maintained at 25°C in the dark and examined daily from 4 days after plating for 14 days. Conidia that had the characteristic morphologies of *Metarhizium* were transferred to PDA plates (4 g l^−1^ potato extract, 20 g l^−1^ glucose, 15 g l^−1^ agar, 0.3 g l^−1^ chloramphenicol) and subcultured. If there was more than 1 colony type in a soil sample, the conidia of each type were isolated and separately subcultured.

### Genotyping of *Metarhizium* isolates by PCR–RFLP

Crude DNA samples were prepared as following. Mycelia from a pure culture on an agar plate were removed with a sterile micropipette tip and suspended in 50 µl of Tris–EDTA (TE) buffer containing RNase A (10 µmol l^−1^ pH 8.0 Tris–HCl, 1 µmol l^−1^ pH 8.0 EDTA, 0.01% RNase A (w/v)). The suspensions were frozen at least once before being used for PCR. The crude DNA solutions were kept at −20°C.

For some fungal isolates, approximately 50 mg of mycelia was homogenized in DNA extraction buffer (4 mmol l^−1^ Tris–HCl, 250 mmol l^−1^ NaCl, 25 mmol l^−1^ EDTA, 1% SDS (w/v)) and extracted with an equal volume of phenol/chloroform/isoamyl alcohol (25:24:1) for 10 min on a vortex mixer. The solution was then centrifuged at 17,000 × *g* to separate the phases, and the upper aqueous phase was transferred to a 1.5-ml tube. DNA was precipitated from the solution by the addition of approximately 0.8 volumes of isopropanol, washed with 70% ethanol, dried under vacuum, and resuspended in TE buffer containing RNase A. The DNA solutions were kept at 4°C or −20°C.

The primers used for the amplification of the 5′-EF1-α were EF1T and EF2T (Table 2). PCRs were performed in 10-µl reaction volumes, which contained 2–20 ng of genomic DNA or 10% (v/v) crude DNA solution, 1× reaction buffer for KOD FX Neo (TOYOBO, Japan), 0.5 µmol l^−1^ of each primer, and 0.1–0.2 U KOD FX Neo (TOYOBO, Japan). The conditions used for PCR amplification were 2 min at 94°C, followed by 38 cycles for 10 s at 98°C, 30 s at 52°C, and 30 s at 68°C.

**Table 2.** Primers used for PCRs in this study.

Loci	Primers	Sequences (5ʹ-3ʹ)	References
5ʹEF1-α	EF1T (F)	TTTGCGAAGATGCGTTGAAG	Rehner and Buckley 2005
	EF2T (R)	CCGCTGCTTACCTGATTGTG	Rehner and Buckley 2005
rDNA IGS	IGS28S4 (F)	CCTTGTTGTTACGATCTGCTGAGGG	Pantou et al. 2003
	IGS18S4 (R)	TAATGAGCCATTCGCAGTTTCGCTG	Pantou et al. 2003

PCR amplifications of the rDNA IGS region were also performed in the same manner except that IGS28S4 and IGS18S4 (Table 2) were used as primers and the PCR conditions were 38 cycles for 10 s at 98°C, 30 s at 63°C, and 90–120 s at 68°C.

The PCR products of 5′-EF1-α were digested with *Mlu*CI or Tsp509I (NEB, Japan). The PCR products of rDNA IGS were digested with *Hae*III (NEB, Japan). Restriction digestion was performed in volumes of 20 µl comprising 9 µl of the PCR product, 1× restriction enzyme buffer, 1–3 units of the respective restriction enzyme, and sterile distilled water. Reaction mixtures were incubated at 37°C for 12–16 h. Electrophoresis of 5–6 µl of each of the digested samples was performed on 2% Agarose 21 (Nippon Gene, Japan) with a 50-bp size marker at 100 V for 30–60 min in 1× Tris–borate EDTA buffer. The DNA fragments in the gel were visualized under ultraviolet light after staining the gel with ethidium bromide.

### Species identification by molecular phylogenetic analysis of DNA sequences

DNA sequencing of 5′-EF1-α and species identification by phylogenetic analysis of the DNA sequence data was conducted as described by Nishi et al. (2013). A region of 5′-EF1-α-like DNA sequence in the whole genome shotgun sequence of *Pochonia chlamydosporia* 123 (Genbank ID: AOSW01006373, range: 531–1400) was used as an outgroup. The multiple alignments and phylogenetic tree were deposited in TreeBASE (http://treebase.org) as S16497.

### Analysis of the association between habitat types and species in the population of *Metarhizium* spp. in the Mt. Tachibana-yama area (Fukuoka, Japan)

A total of 82 soil samples were collected in the Mt. Tachibana-yama area (Fukuoka, Japan) (Table 1). The longitude and latitude of sampling sites were measured with a GPS navigation device (eTrex Summit HC: Germin, Switzerland). The sampling sites were plotted on the maps illustrated on the basis of satellite images.

*Metarhizium* spp. were isolated from soil samples in the same manner as described above. The isolates were classified by PCR–RFLP genotyping of 5′-EF1-α and rDNA IGS. Differences in the frequency of detection of each genotype or species were evaluated by Fisher’s exact test.

## Results

### Isolation of *Metarhizium* from soil samples

A total of 279 soil samples were collected from areas in Japan located from 26°N to 46°N (Table 1). Out of the 197 soil samples shown in Table 1 (82 soil samples collected from the Mt. Tachibana-yama area in Table 1 were separately analysed), fungi identified as *Metarhizium* spp. by morphology (cylindrical conidia produced in chain) were detected from 129 soil samples (detection rate = 65.5%). The detection rate of *Metarhizium* spp. in soil samples from the Mt. Tachibanayam area was 78.9%, which was significantly higher than the detection rate in other soil samples (Fisher’s exact test; *p = *0.01493 < 0.05). A total of 302 isolates from the total soil samples in Table 1 were subcultured and used for the following analyses.

### Genotyping by PCR–RFLP of 5′-EF1-α and rDNA IGS

**Figure 1.**
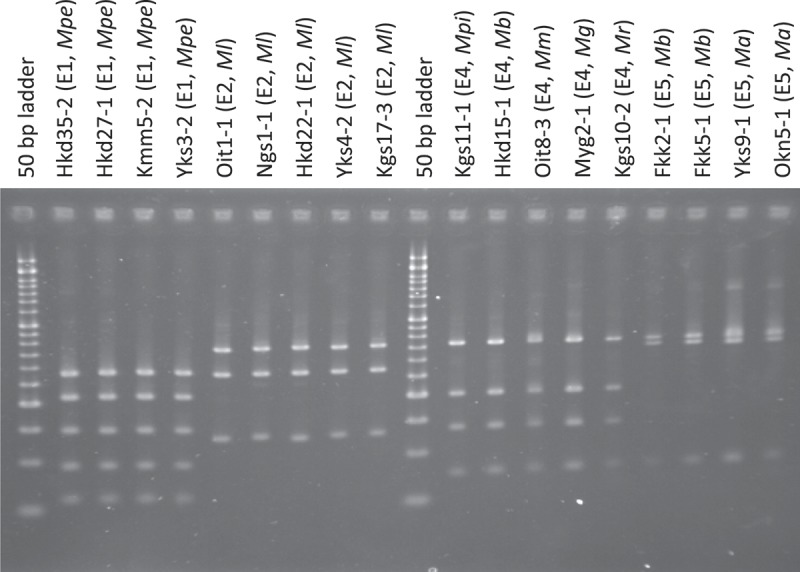
PCR–RFLP profiles of *Metarhizium* spp. (amplicons of 5′-EF1-α digested with *Mlu*CI). The size marker consists of 17 fragments between 50 and 1200 bp (every 50 bp up to 500 bp, every 100 bp from 500 bp to 1000 bp, 1200 bp, and 1500 bp). The characters in parentheses after the strain name indicate the genotypes of PCR–RFLP. The names of *Metarhizium* spp. are abbreviated as follows: *Ma, M. anisopliae; Mb, M. brunneum; Mpe, M. pemphigi; Mg, M. guizhouense; Ml, M. lepidiotae; Mm, M. majus; Mpi, M. pingshaense; Mr, M. robertsii*.

PCRs with primer EF1T and EF2T amplified a single region (approximately sized 800 bp) for all isolates. All isolates were classified into four groups by the length of the products digested with *Mlu*CI (E1, E2, E4, and E5; Figure 1).

**Figure 2.**
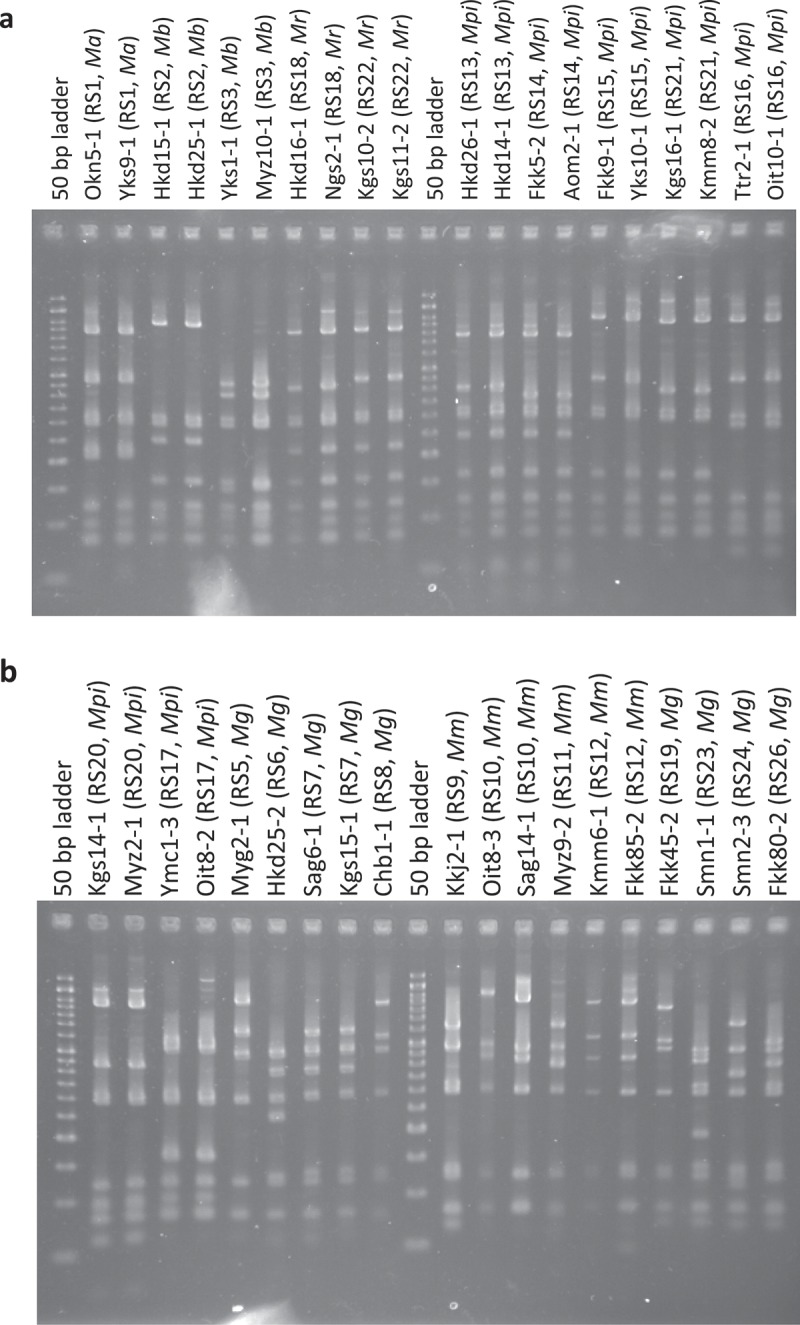
PCR–RFLP profiles of *Metarhizium* spp. (amplicons of rDNA IGS digested with *Hae*III). a. Profiles of 20 isolates that are classified into 10 genotypes (RS1–3, 13–16, 18, 21, 22). b. Profiles of 19 isolates that classified into 14 genotypes (RS5–12, 17, 19, 20, 23, 24, 26). The size marker consists of 17 fragments between 50 and 1200 bp (every 50 bp up to 500 bp, every 100 bp from 500 bp to 1000 bp, 1200 bp, and 1500 bp). The characters after the isolate names indicate the genotypes of PCR–RFLP and species identified by the 5′-EF1-α sequences. The names of *Metarhizium* spp. are abbreviated as follows: *Ma, M. anisopliae; Mb, M. brunneum; Mg, M. guizhouense; Mm, M. majus; Mpi, M. pingshaense; Mr, M. robertsii.*

PCRs of the rDNA IGS region of the isolates belonging to E4 and E5 resulted in the amplification of a single region. On the other hand, PCRs targeted towards the same region of the isolates belonging to E1 and E2 resulted in the amplification of more than 1 band. Some different primer pairs or PCR programmes were used for the isolates of E1 and E2, but they did not provide single band amplification. Thus, the amplicons of the isolates of E4 or E5 were digested with *Hae*III. Twenty-four length polymorphisms (RS1–RS3, RS5–RS24, and RS26) were recognized in the restriction patterns (Figure 2). The most common genotypes were RS13 and RS14, which accounted for 24.2% (46/190) and 12.6% (24/190) of the total isolates, respectively, excluding the isolates from the Mt. Tachibana-yama area.

### Species identification by phylogenetic analysis of the 5′-EF1-α sequences

The 5′-EF1-α regions of 139 representative isolates from the 26 genotypes (E1, E2, RS1–RS3, RS5–RS24, and RS26) were sequenced and the data sets were reduced to 60 sequences by eliminating duplicated data. Molecular phylogenetic analysis of the 60 sequences and 13 reference sequences from Genbank showed that isolates from the 26 genotypes belonged to eight known species of *Metarhizium: M. anisopliae* (*Ma*), *Mb, M. guizhouense* (*Mg*), *M. pemphigi* (*Mpe*), *M. lepidiotae* (*Ml*), *M. majus* (*Mm*), *M. pingshaense* (*Mpi*), and *Mr*. The multiple alignments and phylogenetic tree were deposited in TreeBASE (S16497). Table 3 shows the numbers of isolates that were identified by DNA sequence analysis of 5′-EF1-α.

**Table 3.** The number of the isolates belonging to each PCR-RFLP genotypes or species.

Genotypes of the 5ʹEF1-α	Genotypes of the rDNA IGS	Number of isolates (number of isolates whose 5ʹEF1-α sequences were determined)	Species corresponding to genotypes^a^
E1	**–**	29 (15)	*Mpe*
E2	**–**	10 (10)	*Ml*
E4 or E5	**RS1**	6 (5)	*Ma*
	**RS2**	8 (7)	*Mb*
	**RS3**	39 (10)	*Mb*
	**RS5**	3 (2)	*Mg*
	**RS6**	1 (1)	*Mg*
	**RS7**	3 (2)	*Mg*
	**RS8**	8 (2)	*Mg*
	**RS9**	4 (2)	*Mm*
	**RS10**	2 (2)	*Mm*
	**RS11**	1 (1)	*Mm*
	**RS12**	4 (3)	*Mm*
	**RS13**	61 (26)	*Mpi*
	**RS14**	61 (10)	*Mpi*
	**RS15**	20 (9)	*Mpi*
	**RS16**	3 (3)	*Mpi*
	**RS17**	3 (3)	*Mpi*
	**RS18**	14 (10)	*Mr*
	**RS19**	2 (1)	*Mg*
	**RS20**	9 (6)	*Mpi*
	**RS21**	5 (3)	*Mpi*
	**RS22**	2 (2)	*Mr*
	**RS23**	2 (1)	*Mg*
	**RS24**	1 (1)	*Mg*
	**RS26**	1 (1)	*Mg*
Total		302 (138)	

^a^*Metarhizium* spp. are abbreviated as follows: *Ma, M. anisopliae; Mb, M. brunneum; Mg, M. guizhouense; Mm, M. majus; Mpe, M. pemphigi; Mpi, M. pingshaense; Mr, M. robertsii*.

The PCR–RFLP genotypes corresponded to or subdivided each species; therefore, all isolates belonging to the same PCR–RFLP genotype belonged to the same species. Among the isolates that were identified by phylogenetic analysis of the DNA sequence of 5′-EF1-α, isolates grouped as E1, E2, and RS1 corresponded to *Mpe, Ml*, and *Ma*, respectively. The other 5 species corresponded to more than 1 genotype: 2, 8, 4, 7, and 2 genotypes corresponded to *Mb, Mg, Mm, Mpi*, and *Mr*, respectively (Table 3). The PCR–RFLP genotypes of rDNA IGS were, to some extent, correlated with intraspecific phylogenetic genealogies (data not shown).

## Species association of Metarhizium spp.

**Figure 3.**
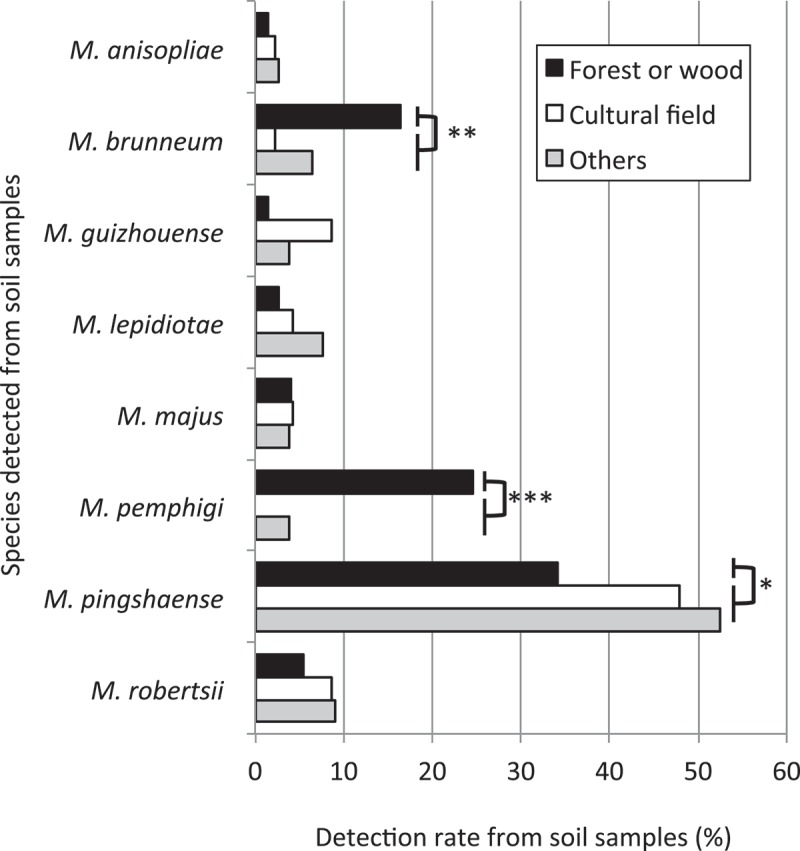
Detection rates of eight *Metarhizium* spp. from soil samples collected from different habitat types (Fisher’s exact test; *: *p* < 0.05, **: *p* < 0.01, ***: *p* < 0.001).

All isolates were identified on the basis of the combination of PCR–RFLP genotyping and phylogenetic analysis of 5′-EF1-α sequences (Table 3). Figure 3 shows the detection rate of the eight species from the soil samples. The isolates from the Mt. Tachibana-yama area were excluded from this analysis. The result showed that *Mpi* was the most common species and accounted for 54.2% of the total isolates. *Mpi* contains the two largest genotypes, RS13 and RS14, which accounted for 36.8% of the total isolates.

The three most frequently detected species, *Mb, Mpe*, and *Mpi*, displayed different habitat preferences. *Mb* and *Mpe* were detected from soil samples from forests or woods at a frequency that was significantly greater than that of agricultural fields or other habitats (one-sided Fisher’s exact test: *p = *0.0075 (*Mb*); *p* = 1.9 × 10^−6^ (*Mpe*)). On the other hand, *Mpi* was significantly frequently detected from soil samples from agricultural fields or other habitats than from forest or wood habitats (one-sided Fisher’s exact test: *p = *0.017). *Mpi* was the most frequently detected species across all three habitat types.

**Figure 4.**
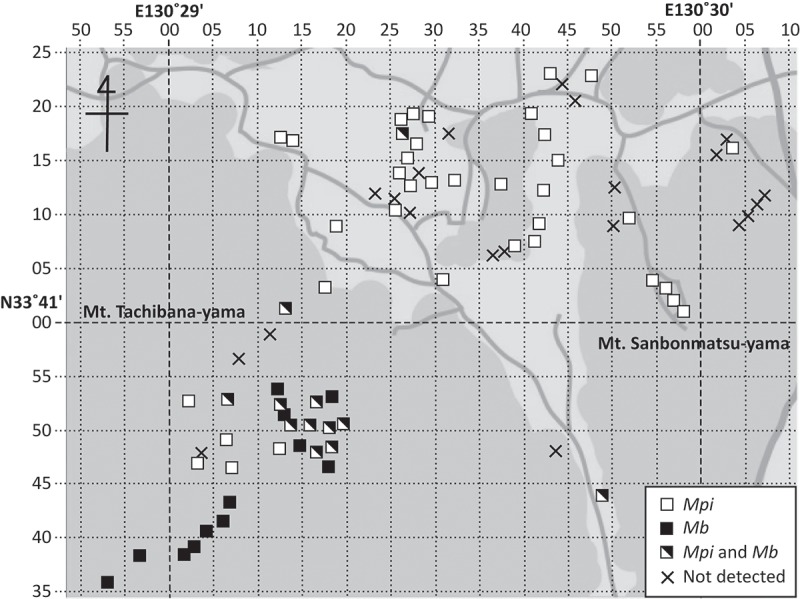
Sample sites of soils from which *Metarhizium brunneum* (*Mb*) and *M. pingshanese* (*Mpi*) were detected in an area of heterogeneous habitat around Mt. Tachibana-yama, Fukuoka, Japan. Grey areas were considered to be rich in trees.

**Figure 5.**
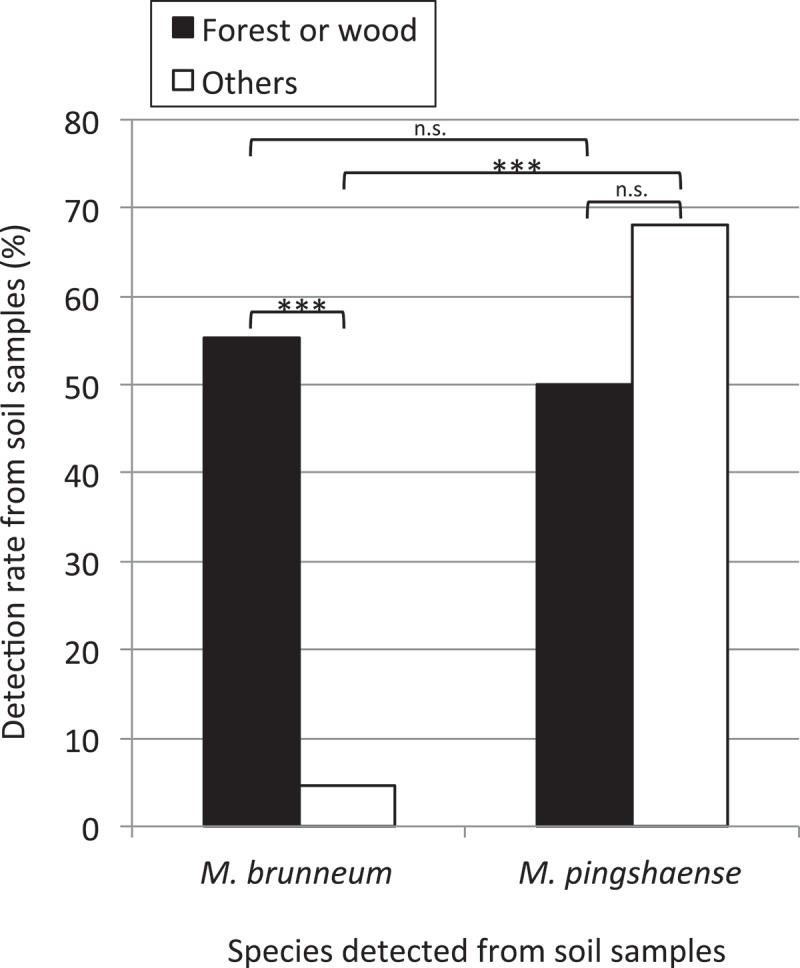
Detection rates of *Metarhizium brunneum* and *M. pingshaense* from soil samples in the Mt. Tachibana-yama area (Fisher’s exact test; *: *p* < 0.05, **: *p* < 0.01, ***: *p* < 0.001, n.s.: *p* > 0.05).

A total of 14 genotypes of *Metarhizium* spp. were detected from the 82 soil samples intensively collected from the Mt. Tachibana-yama area (Fukuoka, Japan) (Table 4). The 14 genotypes belonged to 7 species (*Ma, Mb, Mg, Mm, Mpe, Mpi*, and *Mr*). *Mpi* was the most common species detected in the area (detection rate = 59.8% (49/82)), followed by *Mb* (detection rate = 28.0% (23/82)). With regard to spatial distribution in the Mt. Tachibana-yama area, *Mpi* (RS13, 14, 15, 20, 21) appeared to be evenly distributed in various environments, whereas *Mb* (RS3) appeared to be restricted to forest soil (Figure 4). *Mpe* (E1) and *Mg* (RS5, 8, 19, 26) in the area also appeared to be restricted to forests (Table 4). Compared with *Mpi*, the number of forest soil samples from which *Mb* was detected was not significantly different (two-sided Fisher’s exact test: *p = *0.13 > 0.05), although the number of soil samples from the other habitat types from which *Mb* was detected was significantly lower (one-sided Fisher’s exact test: *p = *1.1 × 10^−10^ < 0.001) (Figure 5). *Mb* was significantly more frequently detected in soil samples from forest habitats than from other habitat types (one-sided Fisher’s exact test: *p* = 2.1 × 10^−7^ < 0.001), whereas there was no significant difference in the frequency of detection of *Mpi* between forest habitats and other habitat types (one-sided Fisher’s exact test: *p = *0.073 > 0.05).

**Table 4.** Detection rate of *Metarhizium* isolates of different genotypes from soil samples of the Tachibana-yama area.

Genotypes of the rDNA IGS	Detection rate (%)		
	Forest or wood	Others (including cultural field)	Total
E1	18.4	0.0	8.5
RS1	5.3	0.0	2.4
RS3	55.3	4.5	28.0
RS5	5.3	0.0	2.4
RS8	15.8	0.0	7.3
RS12	7.9	0.0	3.7
RS13	7.9	25.0	17.1
RS14	42.1	47.7	45.1
RS15	5.3	9.1	7.3
RS18	0.0	2.3	1.2
RS19	2.6	2.3	2.4
RS20	0.0	4.5	2.4
RS21	2.6	2.3	2.4
RS26	2.6	0.0	1.2

## Discussion

In the current study, soil isolates of *Metarhizium* spp. in Japan from a previous study of Nishi et al. (2011) and additional soil isolates were analysed in terms of species composition and habitat preferences. The results of the current study revealed the following: (1) 302 Japanese isolates were classified into 26 genotypes by PCR–RFLP of 5′-EF1-α and rDNA IGS and were identified as eight species by DNA sequence analysis of 5′-EF1-α; (2) *Metarhizium* spp. isolated from soil samples in Japan (mainly from the Kyushu district located 31°N to 34°N) showed highly biased species compositions, and *Mpi* was the most common species, which comprised approximately 50% of the total isolates; (3) *Mpi* was detected from soil samples from various environments, whereas *Mb* and *Mpe* were detected preferentially from forest environments; (4) in an intensive investigation in the Tachibana-yama area (approximately 1.6 km × 1.0 km) in Fukuoka, Japan, seven *Metarhizium* spp. composed of 14 genotypes were detected, and species composition and habitat preferences in this area were similar to the results obtained throughout Japan. The current study is the first to report the high abundance of *Mpi* in Japan and the significant difference of its habitat preference from *Mb* and *Mpe*.

To identify the species of the 302 soil isolates of *Metarhizium* spp. in Japan, we utilized PCR–RFLP of 5′-EF1-α and rDNA IGS as the first grouping method. DNA sequences of 5′-EF1-α were phylogenetically analysed for species identification for the representative isolates of each genotype of PCR–RFLP. PCR–RFLP of 5′-EF1-α with *Mlu*CI digestion distinguished isolates of *Mpe* and *Ml* and the other monophyletic group including six species (*Ma, Mb, Mg, Mm, Mpi*, and *Mr*). In combination with PCR–RFLP of 5′-EF1-α and digestion with some other restriction enzymes, some pairs among the six species were also distinguished from each other as per the method used by Wyrebek et al. (2011): their PCR–RFLP analysis of *Metarhizium* spp. in eastern Canada successfully distinguished *Mb, Mg*, and *Mr*. In the current study, however, it was difficult to distinguish the Japanese isolates of *Mr* and *Mpi* by PCR–RFLP of 5′-EF1-α. This is because of minor variations of the sequence of 5′-EF1-α between *Mpi* and *Mr*. However, high polymorphisms were detected among the six species, including between *Mpi* and *Mr* by PCR–RFLP of rDNA IGS. PCR–RFLP of 5′-EF1-α and rDNA IGS appears to be beneficial for first grouping of a large number of *Metarhizium* isolates.

We observed high abundance of *Mpi* in soils in Japan. *Mpi* was the most frequently detected species across all habitats (forest or wood habitat, agricultural field, and others habitats, Figure 3). This finding is similar to the results found in western Canada, where two closely related genotypes were predominantly distributed in various habitat types (the species of the two genotypes were unknown) (Inglis et al. 2008). According to the studies of *Metarhizium* spp. in other countries, the predominant genotype or species of *Metarhizium* spp. in soil appears to vary geographically. In Brazil, *M. anisopliae* sensu strict was most abundant in soil (Rocha et al. 2013). In eastern Canada, *Mr* was abundant in the rhizosphere soil of wild flowers (Wyrebek et al. 2011). In New Zealand, *M. novazealandicum* (*M. flavoviride* var. *novazealandicum*) was reported to be dominant in soil (Driver et al. 2000). Although the habitat types of soil samplings should be considered when discussing the dominant species, because some groups of *Metarhizium* spp. show clear habitat preferences (Bidochka et al. 2001), it appears that *Mpi* is not as abundant in those research areas as in Japan, because *Mpi* was most frequently detected in both forest and agricultural environments in Japan. The reason for these geographical differences is as yet unknown. Wang et al. (2011) suggested the importance of the abilities of root colonization and stress resistance rather than virulence against insects in the persistence of an introduced strain of *Mr* in a test field. According to their results, the difference in the composition of genotypic groups may also be associated with the difference in rhizosphere competency and response against environmental stresses such as temperature and sunlight. Among *Metarhizium* spp., *Mr* is relatively well studied as a root colonizer (Hu and St. Leger 2002; Wyrebek et al. 2011). *Mpi* may also be a root colonizer considering its wide distribution in soil in Japan and its phylogenetically close relationship with *Mr*. The predominance of *Mpi* across habitats has implications for the efficacious development of this species as a microbial control agent of insect pests in Japan. Further studies regarding the characterization of *Mpi* is important to determine the factors driving the abundance of *Mpi* in Japan.

Compared with *Mpi*, the distributions of *Mb* and *Mpe* in Japan were significantly restricted to forest environments. This habitat preference was indicated in two previous studies (Nishi et al. 2011, 2013) and was confirmed in the current study. A comparison of *in vitro* germination of conidia demonstrated that the two species showed a higher germination rate under cold conditions and a lower germination rate under hot conditions compared with the other six species including *Mpi* (Nishi et al. 2013). According to these results, this cold-adapted characteristic appears to restrict their distribution to forest or wood habitats. In eastern Canada, *Mb* was associated with the rhizosphere soil of trees rather than with the rhizospheres of plants in open fields (Wyrebek et al. 2011), indicating the association of *Mb* with forested habitat. Furthermore, the cold-active and forest-dominant genotypic group of *M. anisopliae sensu lato* had been reported in the same area (Bidochka et al. 2001), which partially corresponds to *Mb* (Bischoff et al. 2009). Accordingly, *Mb* in Canada and Japan seem to have similar habitat and temperature preferences. However, in Japan, the most common species in forested areas was not *Mb* but *Mpi*, which was also predominant across all habitat types (Figure 3).

In contrast to eastern Canada and Japan, genotypes of *M. anisopliae sensu lato* in Chile, Turkey, and western Canada (British Columbia) were not associated with habitat types (Velasquez et al. 2007; Inglis et al. 2008; Sevim et al. 2012). In the current study, the habitat preference of *Metarhizium* spp. other than *Mb* and *Mpe* was not clearly determined even if species associations were analysed by subdivided habitat types (paddy field, riverside, grass field, groves, bushes, etc., data not shown). Accordingly, the unclear habitat preferences in the three countries may be partly caused by the low frequency of isolates of *Mb* and *Mpe* in the isolate of their analyses. Another possible reason is the predominance of some genotypic groups across different habitat types in their analysis. In fact, in the research in western Canada (Inglis et al. 2008), two genotypes (genotype 1.1 and 1.2) detected from various habitat types covered 89.1% of the total isolates.

The intensive research in the Mt. Tachibana-yama area (1.6 × 1.0 km) showed the existence of 14 genotypes (corresponding to seven species) in this area. The results demonstrate that various genotypes can live in close spatial proximity to each other. This area may be especially abundant in *Metarhizium* spp. because the detection rate of *Metarhizium* in this area was higher than that throughout Japan (Table 1). This number of species is large compared with that found in other studies in the other countries: three species were detected from soils in eastern Canada and Brazil, respectively (Wyrebek et al. 2011; Rocha et al. 2013). It is not known how such a large number of genotypic groups can exist in close spatial proximity to each other. It may be explained by the ecological differences among the genotypic groups, although the fact that some groups prefer forest habitats while others do not is clearly not enough to elucidate the differences. These groups may vary in their association with plant species considering that three species of *Metarhizium* in eastern Canada associated with the rhizosphere of specific plant types (Wyrebek et al. 2011).

In conclusion, we observed that *Mpi* predominated in both agricultural and forest soils in Japan and that *Mb* and *Mpe* were, in contrast, restricted to forest soils. The observation of *Mpi* predominance across different habitats in the current study is similar to a previous finding in western Canada (British Columbia) where two closely related genotypes are predominant across different habitats (Inglis et al. 2008). The restriction of *Mb* and *Mpe* to forest habitats in the current study is partly comparable to the findings of a previous study in eastern Canada in which two genotypic groups separately occupied agricultural and forest environments (Bidochka et al. 2001). We also observed high genetic diversity of *Metarhizium* in a small area. *Metarhizium* spp. in Japan are genetically very diverse and appear to form complex species associations compared with those in Canada. Further field studies focusing on ecological differences among species or genotypic groups of *Metarhizium* are necessary to understand how this genetic diversity is maintained in *Metarhizium* spp. in Japan.
